# Overview of the Metallization Approaches for Carbyne-Based Devices

**DOI:** 10.3390/molecules28176409

**Published:** 2023-09-02

**Authors:** Rade Tomov, Mariya Aleksandrova

**Affiliations:** Department of Microelectronics, Technical University of Sofia, 1000 Sofia, Bulgaria; rtomov@tu-sofia.bg

**Keywords:** metallization, carbyne, carbon chain, metal interface, electrical contact

## Abstract

Metallization for contacts in organic electronic nanodevices is of great importance for their performance. A lot of effects can appear at the contact/organic interface and modify the contact parameters, such as contact resistance, adhesive strength, and bonding ability. For novel materials, it is important to study the interactions with metal atoms to develop a suitable technology for contacts, fulfilling to the greatest extent the above-mentioned parameters. A novel material is carbyne, which is still under intensive research because of its great potential in electronics, especially for sensing applications. However, the most appropriate metallization strategy for carbyne-based devices is still unknown, so the interactions between carbyne and metal films should be studied to more precisely direct the development of the metallization technology, and to form contacts that are not limiting factors for device performance.

## 1. Introduction

**Figure 1:**
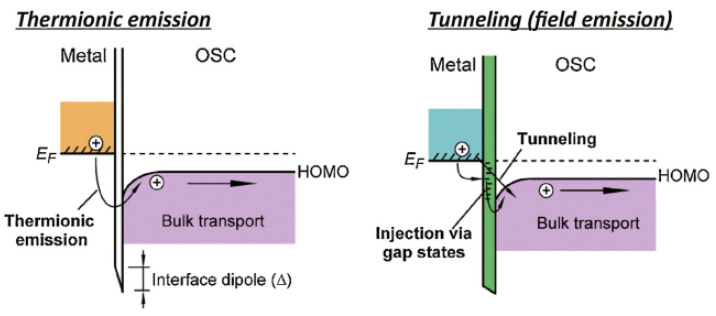
Metal–organic interface and energy level alignments: transport mechanism of the charge carriers by (left) thermionic emission and (right) tunneling [9]. Reprinted from Materials Today, Vol 18, Chuan Liu et al., Contact engineering in organic field-effect transistors, Pages 79–96, Copyright (2015), with permission from Elsevier.

Metallization of a device represents metal layers that have the function of making electrical and mechanical contact with the active material, realizing a specific function. For example, it can be a connection between the sensitive material in sensors and the electrical circuits which collect and process the signals, or in solar cells, to collect the photogenerated charge carriers from the semiconductor and transfer it to the power processing circuit [1,2]. It is important that the metallization is not a bottleneck for the functionality of the device, for example, to not introduce a resistance between the active layer and the metal layer, called contact resistance, across which a significant portion of the energy will be lost as a voltage drop, and the signal will not be transferred to the measuring system. Metallization is important for every type of electronic device, especially for sensors in the field of environmental monitoring or human health care, for example for toxic substances [3], or for the detection of cancer by breath [4], where maximum possible sensitivity is needed. Other examples are energy harvesting systems where lower contact resistance and trapped carriers, or the absence of a potential barrier means higher generated charge carrier extraction and higher efficiency [5]. In the integrated circuits, the type of contact should also provide linear current–voltage characteristics, or if the threshold voltage is required, it should be lower than 0.3 V to reduce device consumption [6]. The requirements that metallization has to meet are low contact resistance, no charge carrier trap formation during deposition [7], good adhesion to the underlying materials, good bondability (the possibility to make reliable bonds with the metal layer with bonding wire), and low diffusion coefficients into the neighboring material. Otherwise, the parameters of the active layer will be uncontrollably modified by the diffused metal atoms in the bulk. In metallization of organic materials, it is important to avoid overheating the organic layers because they are thermally sensitive, and can degrade at elevated temperatures (normally above 130–140 °C) in contrast to their inorganic counterparts [8]. The specific charge carrier transport mechanisms determine the interface behavior with the different metallization systems depending on the energy level alignment between the active organic layer and the metal in direct contact with it (Figure 1) [9].

The transport of the charge carriers across the metal–organic interface can be through thermionic emission, on account of the temperature, or in the case of interface gap states, which are introduced by defects, charge carriers can hop through them across the interface. Fermi level of the metal, $E_{F}$, highest occupied molecular orbital (HOMO), and lowest unoccupied molecular orbital (LUMO) have to be known in contact engineering so that steps such as metal work function modification by introducing a dipole interface layer [9] can be taken if needed. Aside from the typical values of the energy levels of materials being in contact, there are additional factors affecting the energy barrier height, such as the influence of the spin-orbit effects supporting that, in some cases, Schottky barrier heights have metal-induced gap state-derived character [10]. This problem can be partially avoided if inverse structure is fabricated. Thus, another approach for making contact between metals and organic materials is to deposit the organic material on already grown metal electrodes, avoiding exposure of the organic films to extreme temperatures of plasma, electron beam, or another source of high temperature. In this case, the metal layer is already formed, and all the atoms are organized in one of the basic types of crystal structures (polycrystalline or single crystal), or they are amorphous [11].

**Figure 2:**
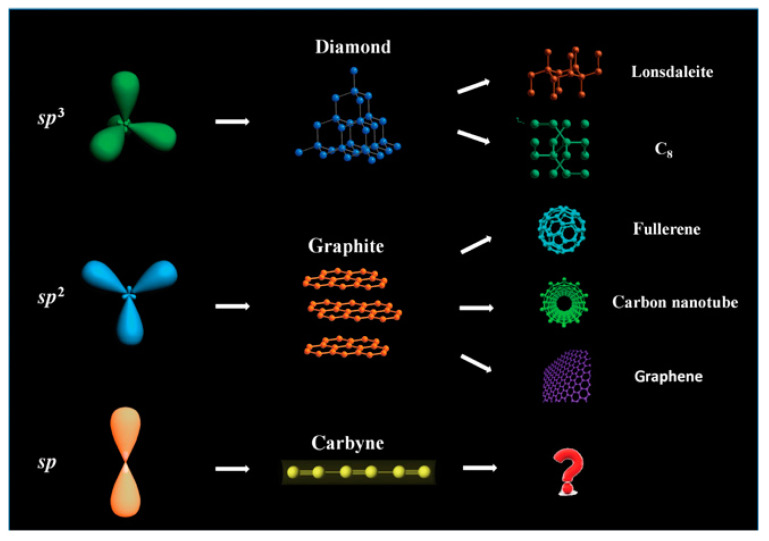
Carbon allotropes [13].

As new nanomaterials have been developed and implemented into advanced electronic devices, new metallization systems have to be explored. This review is motivated by the 1D nanomaterial called carbyne (Figure 2), which is a carbon allotrope consisting of one-dimensional linear chains of carbon atoms with alternating single and triple bonds, which is also called polyyne, or chain with only double bonds between the carbon atoms, also called cumulene [12].

Depending on the types of carbon bonds—cumulene or polyyne—the carbyne can have two types of electrical behavior: either semiconductor or metallic. Regarding the stability, for carbon chains having between 70 and 160 atoms, bond length alteration (BLA) is oscillatory and depends on the number of atoms, but for chains of over 160 atoms, this effect is negligible, and the potential for the carbyne to undergo from cumulenic to polyynic is independent of the chain length. It means that the semiconducting polyynic structure is more stable than the metallic cumulenic [14].

A model of 1D molecular wires connected between electrodes has been reported [15], and the influence of the presence of defects on properties such as conductance and the density of states has been investigated. The numerical calculations have shown that the transport properties are highly sensitive to the positions of the defects. It has been found that the introduction of a single defect in the molecular wire that divides it into two fragments with an odd number of sites creates a new conduction channel at the center of the band gap, resulting in higher zero-bias conductance than for defect-free systems. However, defect formations in the carbyne single chains are the object of molecular engineering, and is related to the chemical synthesis of the material. For this reason, it is out of the scope of the present discussion, which is mainly focused on the interface engineering. When bulk carbyne is in question, the problems of the defects are related to the interfacial states induced after deposition of metal contacts, which are most often of thermal origin.

As carbyne can be used in making gas sensors for volatile toxic compounds [16], it is important to develop the metallization technology so that the sensitivity of the sensors is not limited by the metallization process. A variety of works on interactions between the linear carbon chains, or carbyne with different metals have been studied, some based on simulations, others based on experiments, which will lead to more precise experiment design and ultimately, the best possible (resistance-free and defect-free) metal layer formation for contacts on devices built with carbyne.

Although the researchers still put their efforts into investigating the carbyne material itself from the perspective of microstructural, chemical, physical, and mechanical properties, its advantages are expected to unlock the potential of this material for commercially available devices. As the number of systems using carbyne is still relatively small, it is important that the knowledge about the interaction with basic materials for metallization used in microfabrication technology is summarized. Therefore, in this paper, an overview of the interaction between gold, silver, nickel, copper, and platinum metals with carbyne is discussed. Gold and platinum are expected to form ohmic contact, and silver, Schottky contact. Nickel is very suitable to serve as a barrier layer, but is expected to form an ohmic contact with the carbyne as well, however, at a lower price than gold and platinum. Copper is usually solderable and its presence in a carbyne-based sensor device is expected to give good perspective for easy connectivity to the electrical circuit processing the sensor signals.

## 2. Research Performed on Metal/Carbyne Interfaces

### 2.1. Research Performed on Au/Carbyne Interface

**Figure 3:**
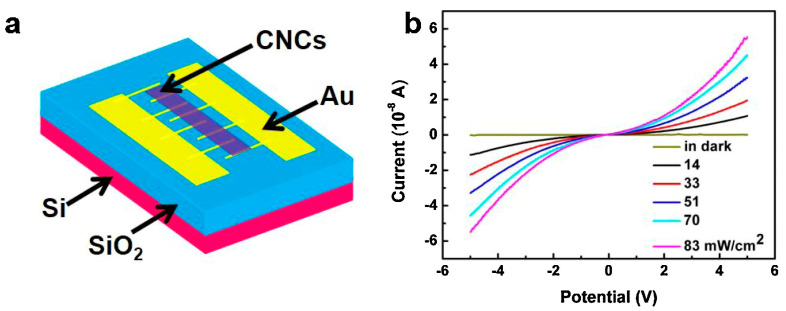
(**a**) Structure of the NO_2_ sensor based on a carbyne sensing layer with Au electrodes, and (**b**) volt–ampere characteristics under different irradiance levels [16]. Reprinted from SENSOR ACTUAT B-CHEM, Vol 316, Fei Yang et al., Visible-light-driven room-temperature gas sensor based on carbyne nanocrystals, Copyright (2020), with permission from Elsevier.

An example of the contact between Au and carbyne nanocrystals, where the carbyne layer has been coated over previously formed Au electrodes, is a gas sensor for NO_2_ molecules (Figure 3). In this device, the detection of analyte molecules has been facilitated by a visible light [16]. Here, photons with a wavelength of 447 nm excite the electrons in the conduction band of the carbyne nanocrystals so that the NO_2_ molecules can accept them. With this, the number of electrons in the conduction band of the carbyne semiconductor decreases and the resistance increases, which is an indication of the presence of NO_2_ molecules. In this work, a Schottky barrier on the contact is referenced as when a depletion region is formed because the work function of the carbyne nanocrystals is lower than Au, and electrons are transferred to the Au electrodes. The gold contacts have been deposited by pulsed laser ablation and formed in clusters, and the carbyne nanocrystals have been prepared by drop coating. Therefore, it is expected that grain boundaries appear between the two materials, rather than continuous layers. Using the vacuum-free process for carbyne deposition seems to affect the oxidation state of the boundaries, and produces a contact barrier at the interface.

**Figure 4:**
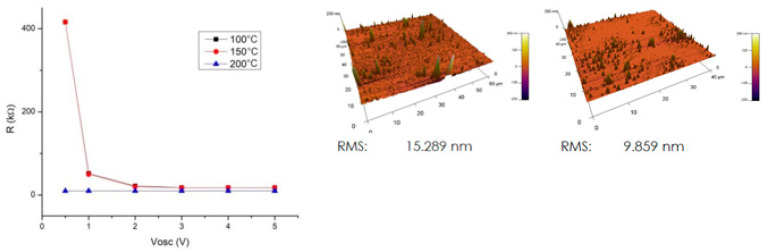
Resistance and surface roughness of the carbyne samples at different temperatures (atomic force microscopy shows the surface before (**left**) and after (**right**) annealing at 200 °C [17]. © [2023] IEEE. Reprinted, with permission, from [R. Tomov and M. Aleksandrova, “Exploring Gold Contacts on Novel Carbyne-enriched Material,” *2023 46th International Spring Seminar on Electronics Technology* (ISSE), Timisoara, Romania, pp. 1–4].

In contrast to these results, an ohmic contact has been reported between vacuum DC sputtered gold continuous films and vacuum ion-assisted pulsed-plasma deposition of carbyne [17]. Gold thin-films have been deposited onto the carbine film. Measurements of the contact resistance of the samples show that annealing at 200 °C (bonding process temperature) of the gold contacts has no negative effect on the interface characteristics, but also decreases the contact resistance and reduces the charge carrier traps, which improves the overall electrical performance of the device. In addition, the surface roughness of the carbyne decreased with the temperature increase, which significantly improves the physical contact between the coatings (Figure 4). Such an interface system is appropriate for surface acoustic wave (SAW) sensor devices, requiring low contact losses for the propagation of the wave without quenching between the input and output transducers. It can be concluded that the Au/carbyne interface is sensitive toward the film deposition methods and postdeposition treatment, which have a strong influence over the behavior of the metal/carbyne state, and can switch the contact type between ohmic and Schottky, accordingly.

**Figure 5:**
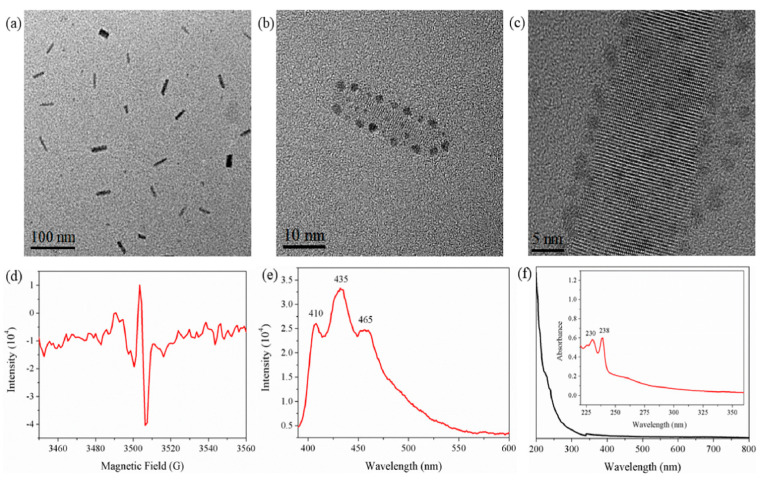
TEM and ESR of carbyne capped with Au nanoparticles [18]. Reprinted from Carbon, Vol 167, Tongming Chen et al. A fluorescent and colorimetric probe of carbyne nanocrystals coated Au nanoparticles for selective and sensitive detection of ferrous ions, Pages 196–201, Copyright (2020), with permission from Elsevier.

Apart from the electrical aspect of this interface, there is also a mechanical aspect, related to the adhesive strength and reliability at possible peeling off, or exfoliation. This issue is of particular importance for wearable devices, built on flexible substrates and periodically subjected to bending or twisting. In the case of carbyne nanocrystals capped with Au nanoparticles by applying laser ablation [18], characterization by transmission electron microscope (TEM) has shown that the Au nanoparticles adhered to the carbyne nanocrystals. The electron spin resonance (ESR) has shown π → π∗ transition, which is an indication for paired intrinsic electrons (Figure 5). TEM images shows a rod-like structure with different width between 10 nm and 30 nm (Figure 5a). The high-resolution TEM images show how the Au nanoparticles adhered to capped carbyne nanocristals (Figure 5b,c). The ESR spectrum in Figure 5d demonstrates a signal of intrinsic unpaired electron in the line shape of Lorentzian. The absorption spectrum of the sample in Figure 5e shows the characteristic peaks, which can be indexed to π→π∗ absorption. The fluorescence spectrum, observed upon excitation of 360 nm in alcohol solvent, shows inherent peaks at purple-blue fluorescence region (Figure 5f). Therefore, these results have demonstrated the application of carbyne/Au interface in dual-functional nanosensors for fluorescent and colorimetric detection of metal ions.

**Figure 6:**
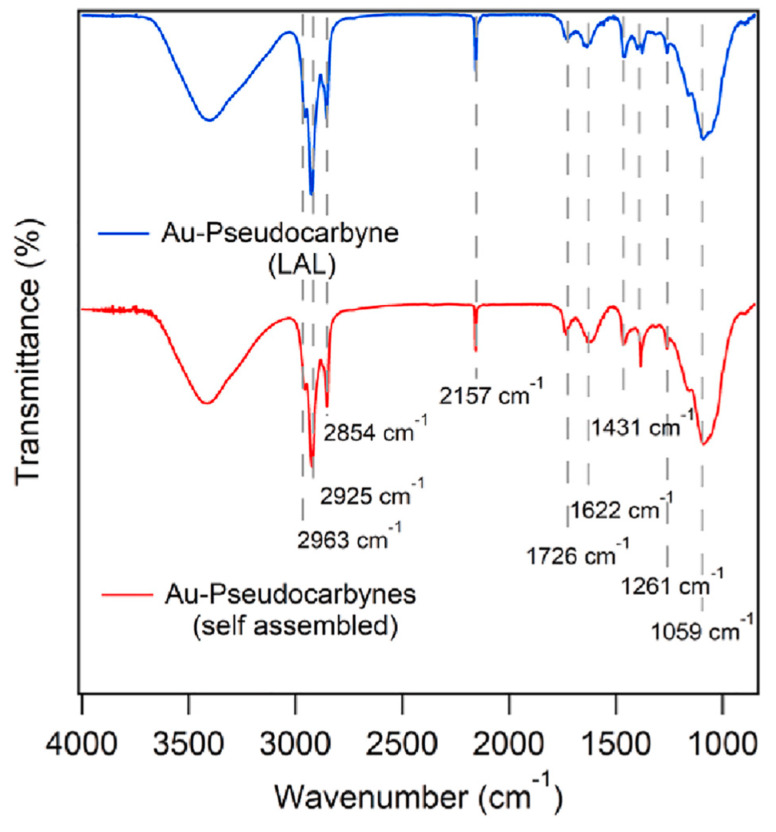
FTIR spectra of Au–pseudocarbynes [19]. Reprinted from Carbon, Vol 205, Hyunsub Kim et al., Formation of Au-pseudocarbynes by self-assembly of carbon chains and gold clusters, Pages 546–551, Copyright (2023), with permission from Elsevier.

A formation of Au–pseudocarbynes via laser ablation (LAL) has been reported [19]. It leads to the hypothesis that when Au is sputtered on a carbyne structure, pseudocarbyne formation is probably at the Au interface, depending on the carbyne microstructure. Fourier transformation infrared spectroscopy (FTIR) measurement is provided as proof of bonded group formation between the gold and polyyne chains (characteristic peak at 2157 cm^−1^—Figure 6).

**Figure 7:**
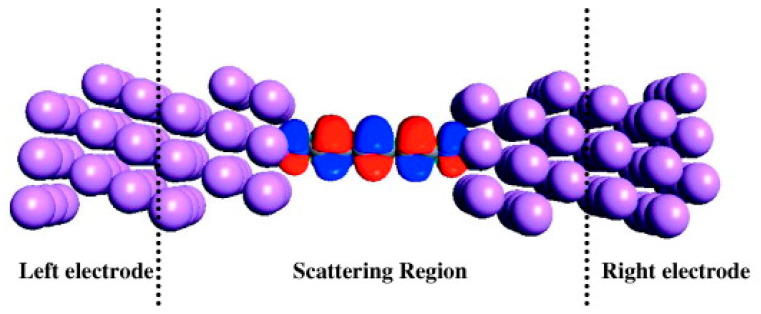
The highest occupied molecular orbital (HOMO) of the nitrogen-capped carbyne sandwiched between Li electrodes [22]. Reprinted from Carbon, Vol 51, Z.H. Zhang et al., Electronic transport of nitrogen-capped monoatomic carbon wires between lithium electrodes, Pages 313–321, Copyright (2013), with permission from Elsevier.

In a study, where polyynes and Au nanoparticles have been prepared by laser ablation, the Au particles have been deposited first, and the polyynes have been grown on them. Surface-enhanced Raman spectroscopy (SERS) measurements have been conducted to compare surfaces with and without the polyynes, and the presence of a band at 2130 cm^−1^ has been shown [20]. In a calculation and simulation study [21], for a chain of carbon atoms contacted via sulfur atoms to gold, the results have shown that the electron density between the carbon atoms in the carbon chain depends on the number of the carbon atoms, and if there are even or odd numbers, chains with even number of carbon atoms conduct less than chains with an odd number. Similar calculations and simulations have been conducted for carbon chains with nitrogen on each side (Figure 7), contacted between Au, Ag, and Cu electrodes, with five and six carbon atoms in the chain.

**Figure 8:**
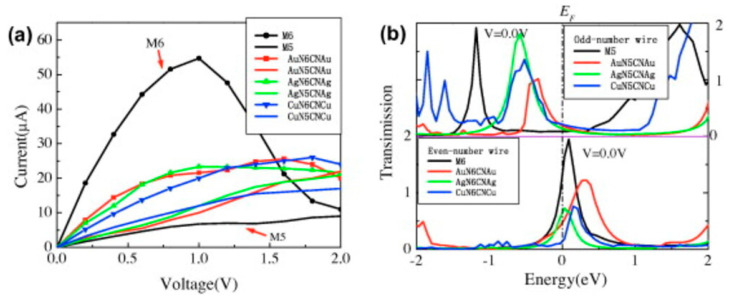
Current–voltage characteristics (**a**) and zero-bias transmission spectra (**b**) of the metal–nitrogen–carbyne–nitrogen–metal sandwich structures. M6 and M5 represent carbon chains consisting of 6 and 5 carbon atoms, respectively [22]. Reprinted from Carbon, Vol 51, Z.H. Zhang et al., Electronic transport of nitrogen-capped monoatomic carbon wires between lithium electrodes, Pages 313–321, Copyright (2013), with permission from Elsevier.

In this case, the chains with six carbon atoms have higher conductivity, both for free-standing and capped by N and metal (Figure 8). This result can be attributed to the N/Li interactions, such as hybridization and overlap of N-capped carbon electron orbital with the Li electron orbital [22].

**Figure 9:**
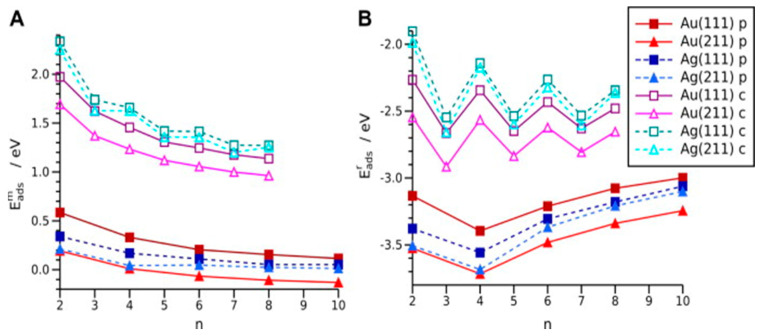
Adsorption energies for cumulenes (c) and polyynes (p) for different numbers of carbon atoms (n) and different surfaces [23]. Reprinted from Carbon, Vol 50, L. Nykänen et al., Computational study of linear carbon chains on gold and silver surfaces, Pages 2752–2763, Copyright (2012), with permission from Elsevier.

**Figure 10:**
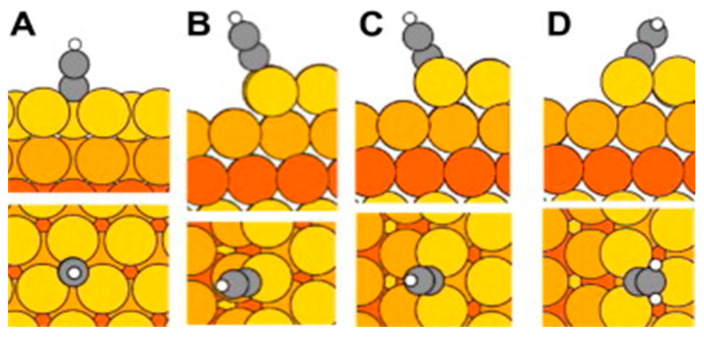
Adsorption places: (**A**) (fcc on 111), (**B**) (top site on 211), (**C**) (bridge site on 211), and (**D**) (hcp on 211). Colors are: carbon—grey, hydrogen—white, metal—orange [23]. Reprinted from Carbon, Vol 50, L. Nykänen et al., Computational study of linear carbon chains on gold and silver surfaces, Pages 2752–2763, Copyright (2012), with permission from Elsevier.

As for interactions between carbyne and gold, in a case of carbyne deposited on gold, an informative study has been performed on calculations of adsorption energies of hydrogen-capped cumulenes and polyynes on gold with different crystal orientations (111 and 211), where the energies were calculated for chains with a different number of carbon atoms (Figure 9), and preferred places for bonding between the carbyne and the metal surface (Figure 10) [23]. The dissociative adsorption of polyynes and cumulenes was investigated on gold and silver surfaces. The adsorption energies were depicted by *E^m^_ads_*, and the energies, corresponding to the metal–chain interaction strength were depicted by *E^r^_ads_*. As it can be seen, there is a difference between adsorption energies of polyynes and cumulenes in the range 1.5–2 eV in Figure 9A, whereas the difference between interaction strength energies, shown in Figure 9B is smaller. The meaning of the smaller energy difference is greater instability of the cumulene carbyne compared to the polyynyl radicals.

The calculations show that the carbyne chains are charged by electrons from the metal atoms, which implies that the contact should be nonrectifying, or ohmic, as no depletion region should be formed by the transfer of electrons from the metal to the contacting material. Another example of transferring electrons from gold to the carbyne is performed by density functional theory (DFT) calculations, which implies that the molecule, in this case polyyne, is charged with electrons, and formed an ohmic contact [24]. A sigma bond between Au and C has been reported, which is favorable for both charge transport and adhesion of a gold layer on carbon. The bond is between the carbon atom on the end of polymethylene [25], and the covalent bond between Au and C, not for chains of carbon [26].

### 2.2. Research Performed on Ag/Carbyne Interface

DFT calculations have suggested a covalent bond between Ag and polyyne. On Ag(111), surface polyynes and cumulenes are more likely to bond with atoms on face-centered cubic (fcc) sites, while on (211), polyynes will bind on the bridge site and cumulenes on the hexagonal close-packed (hcp) site (Figure 10). It has to be certain what the expected number of atoms per one carbon chain is, as it affects the overall electron distributions and the bonds with the metal [23]. These calculations have shown that the bond between Ag surfaces and the carbon chain, in this case, is longer for about a tenth of an angstrom than the bond between the carbon chain and Au, and also that the silver atoms donate more electrons than gold to the carbon chains.

**Figure 11:**
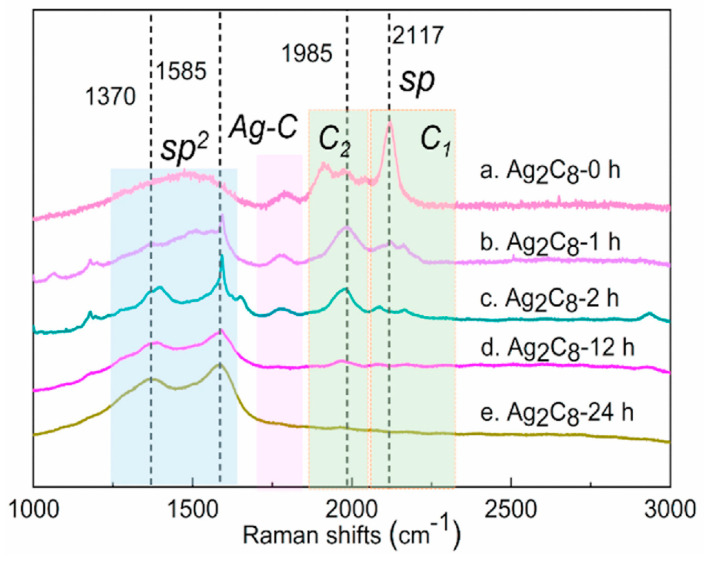
Raman spectra for different storage times of Ag-terminated carbon chains [27]. Reprinted from Carbon, Vol 179, Liang Fang et al., Purification of polyynes via carbides, Pages 28–32, Copyright (2021), with permission from Elsevier.

On the topic of stability under higher temperatures, polyynes on the Ag surface have been annealed and, by the Raman spectra, it is evident that after annealing at 150 °C only the lowered intensity of the shorter polyyne chains were present, while the longer chains have been transformed, increasing the sp2 hybridization in the layer [20]. That is an important fact because depending on the polyyne chains, bonding should be performed at as low as possible temperatures, and measures must be taken to prevent heating of the carbyne layer by adding multiple metal layers which will dissipate the concentrated heat from the wire bonding needle. It has been shown that for Ag-terminated carbon chains, after 24 h of storage, Ag–C bonds fully disappear (Figure 11) [27]. The bonds between the terminated silver atoms and the carbyne chain are made when prepared chemically. It can be an indicator of unstable bonds for metallization purposes, as the linear carbon chains tend to reorganize, so one should a need stronger bond between the metal layer and the carbon chains, leading to improvement of the structure stability.

### 2.3. Research Performed on Ni/Carbyne Interface

**Figure 12:**
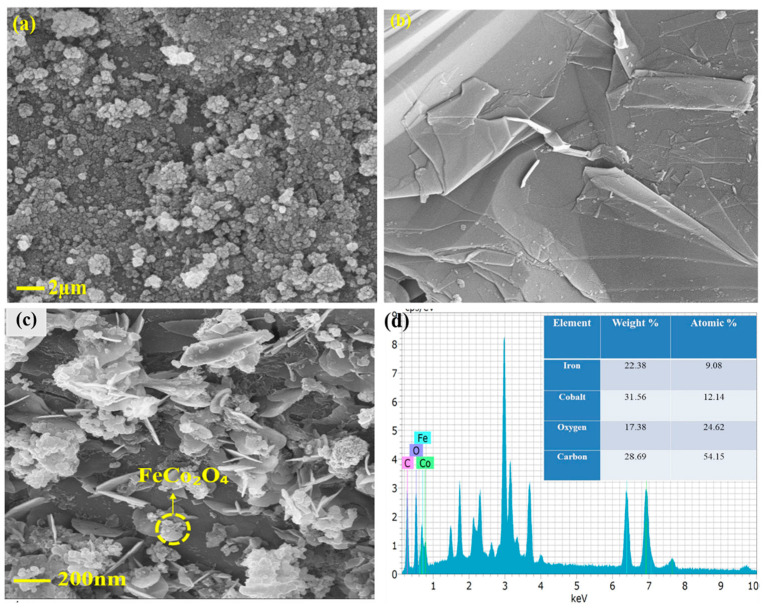
SEM of (**a**) FeCo_2_O_4_, (**b**) carbyne, (**c**) FeCo_2_O_4_@Carbyne nanohybrid on Ni foam and (**d**) EDX of FeCo_2_O_4_@Carbyne [28]. Reprinted from J Energy Storage, Vol 56, Preethi Dhandapani et al., In-situ grown of FeCo_2_O_4_@2D-Carbyne coated nickel foam—A newer nanohybrid electrode for high performance asymmetric supercapacitors, Copyright (2022), with permission from Elsevier.

On the topic of deposition of carbyne on metal layers, for nickel, in a work on carbyne application in supercapacitors, the carbyne that has been grown by chemical reactions on Ni foam has a uniform nanoflake structure (Figure 12) [28].

In another work, also as an electrode for a supercapacitor application, a carbyne-enriched carbon has been grown on a Ni foam, and the results show good electrical conductivity of the electrodes [29].

Simulations on Ni–carbyne nanocomposite show that electrons flow from Ni to carbyne, which affects the electron distribution and results in weaker bonds between the carbon atoms, and likely formations of nickel carbide [30]. Again, the flow of electrons from the metal to the carbyne predicts that the electrical contact in this case should be ohmic.

**Figure 13:**
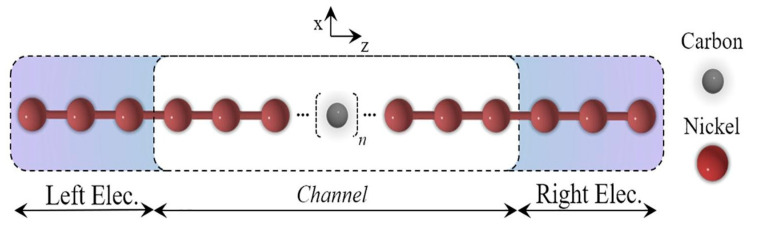
Schematic representation of the simulated structure [31]. Reprinted from MSEB, Vol 262, D.F.S. Ferreira et al., Electronic transport in 1D system with coupling atomic-size nickel electrodes and carbon wires, Copyright (2020), with permission from Elsevier.

Calculations have also been conducted on single carbon wires, contacted by Ni atoms (Figure 13) [31].

**Figure 14:**
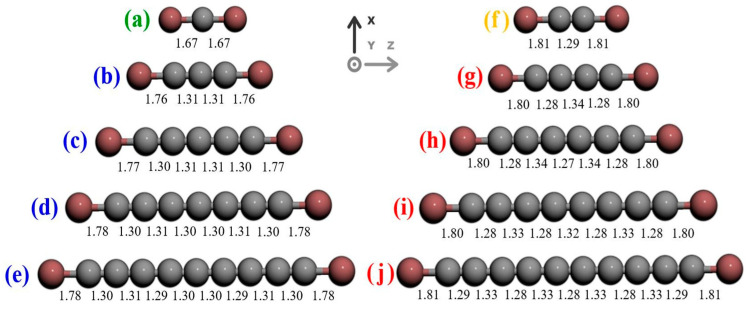
Bond lengths for structures with different numbers of carbon atoms, in angstroms [31]. Reprinted from MSEB, Vol 262, D.F.S. Ferreira et al., Electronic transport in 1D system with coupling atomic-size nickel electrodes and carbon wires, Copyright (2020), with permission from Elsevier.

The bonds between the Ni and C atoms were shorter for carbon wires with an odd number of carbon atoms than for carbon wires with an even number of carbon atoms, and also dependent on the chain length (Figure 14). The length of the single bond Ni-C, shown in Figure 14a is 1.67 Å; (b–e) represents odd chains with a length of approximately 1.77 Å and (f–j) depicts even chains with a length of approximately 1.80 Å. The bond length for (b–e) is typical for a cumulene-type carbyne, and (g–j) is typical for a polyyne-type carbyne.

**Figure 15:**
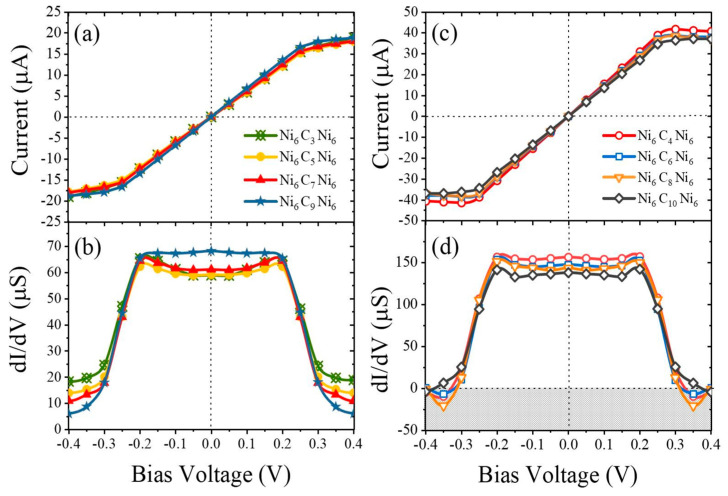
Current–voltage characteristics of Ni–carbyne–Ni structures containing different numbers of carbon atoms [31]. Reprinted from MSEB, Vol 262, D.F.S. Ferreira et al., Electronic transport in 1D system with coupling atomic-size nickel electrodes and carbon wires, Copyright (2020), with permission from Elsevier.

From the current–voltage simulations, it was shown that the structures with an odd number of carbon atoms behave similar to field effect transistors, with the saturation of the current at voltages higher (or lower) than 0.25 V (−0.25 V) (Figure 15a). For comparison, the *I-V* curve for even chains is presented in Figure 15c. Here, the slope of the current-voltage characteristics *dI/dV* for the two cases is very important (Figure 15b,d), as it is related to the carbyne type. Again, it was found that the carbyne chain’s conductivity was different for cases with an even or odd number of carbon atoms, and with increasing the number of carbon atoms, the conductance increases for odd-numbered chains but decreases for even-numbered chains, and even zone with a negative differential resistance occurs (Figure 15d).

In another study, carbyne has been synthesized on a Ni(111) surface, and the carbon chains were present. At interaction between carbon and nickel atoms, the double bonds between the carbon atoms were elongated, but the single bonds stayed the same. When heated to 623 K, or ≈350 °C, Ni–carbide growth was most effective [32], which is another proof that to avoid structural changes in the carbyne layer, and to preserve the carbon chains, care should be taken in the processing and wire bonding to prevent elevation of the temperatures of the layers.

### 2.4. Research Performed on Cu/Carbyne Interface

In a study on a metalated carbyne (reported a chain of Cu atom, followed by two C atoms, repeating), carbyne has been synthesized on Cu (110) with ethyne precursor, where the copper adatoms on the surface replace the hydrogen [33]. In this case, the bonds between the carbon and copper atoms were shown to be 1.902 Å, obtained by DFT calculations. Thus, a possibility of stabilization of the carbyne chains at deposition on Cu substrate is demonstrated.

The calculations showed that there are additional interactions between the carbon chains for carbyne on a copper substrate, explainable by Van der Waals forces, which is another indicator of a change in the stability of the carbyne layer, depending on the substrate [34].

**Figure 16:**
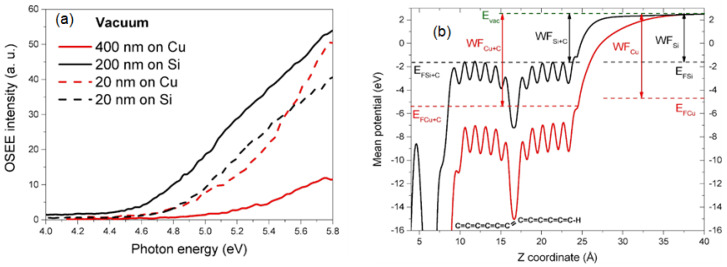
(**a**) Vacuum electron emission (OSEE) spectra, and (**b**) calculated potential diagrams [35]. Reprinted from Carbon, Vol 152, E.A. Buntov et al., Effect of thickness and substrate type on the structure and low vacuum photoemission of carbyne-containing films, Pages 388–395, Copyright (2019), with permission from Elsevier.

The carbyne layer affects the surface on which it is deposited, as has been shown in a study where carbyne films, grown by ion-stimulated condensation from carbon plasma on polycrystalline copper, lower the Fermi level by ~0.9 eV, and this results in a higher work function of the coated copper [35] (Figure 16).

A Raman spectra study of copper-terminated polyyne chains, with a single bond between Cu and C atoms, has shown the presence of a peak at 1960 cm^−1^ and a smaller peak at 2100 cm^−1^. They can be explained by a possible transformation to cumulene chains, with double bonds between the copper and carbon atoms [36,37]. It means that changes are possible in the carbyne chain bonds deposited on a copper substrate, or in the case of copper deposited on a carbyne layer.

### 2.5. Research Performed on Pt/Carbyne Interface

**Figure 17:**
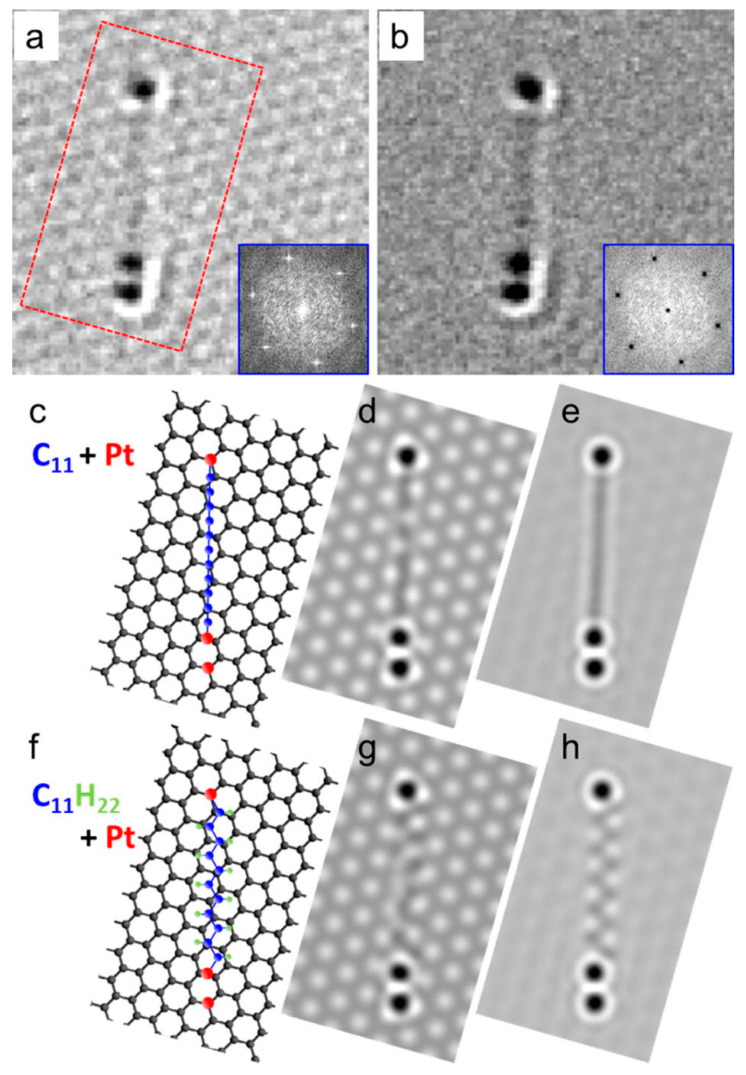
Interaction between carbyne and Pt: (**a**) SEM of Pt-terminated carbyne on graphene, (**b**) removed graphene background by Fourier filtering, (**c**–**e**) simulated straight carbyne, (**f**–**h**) simulated zigzag alkane [38]. Reprinted from Carbon, Vol 80, Emi Kano et al., Direct observation of Pt-terminating carbyne on graphene, Pages 382–386, Copyright (2014), with permission from Elsevier.

In a study of Pt atom deposition by plasma sputtering on graphene, and for the formation of carbyne, it has been concluded that only carbon adatoms participate, and it was confirmed by simulations that the SEM images are of straight carbyne chains terminated by Pt, and not zigzag alkane [38] (Figure 17). The carbyne chains were stable only when terminated by Pt atoms on both sides, otherwise, it bonds with other carbon atoms. These findings can indicate that when carbyne is deposited on Pt layers, the carbyne chains can become terminated and more stable than without Pt atoms.

In DFT simulations on Pt–C_x_–Pt systems, where “x” represents the different number of carbon atoms in the chain, a decrease of the bond length between the Pt and C atoms, with an increase of the carbon chain length from 2.039 Å for a chain with 4 C atoms to 2.031 Å for a chain with 26 carbon atoms, has been calculated [39].

Charge distribution simulations confirmed that the carbon chain length affects the bonds strength between the carbon atoms and Pt atoms.

It should be noted that the calculation and simulation works referenced in Figure 7 (for Au), Figure 16 (for Cu), and Figure 17 (for Pt) have been conducted mostly on a carbyne/metal systems fabricated by top-down and bottom-up approaches, without reporting information about the contact geometry, or to consider wire-type carbyne formation, except for the case of Ni contacts. The results described in Figure 17 showed that the stability of the entire system is also depended on the approach of building the structure, and it is due to chemical interactions between the materials in contact. The dimensionality of the material (nanowire or not) is not a factor in this case.

## 3. Comparison of Systems with Different Metallization

Both ohmic and Schottky contacts between carbyne and Au (Schottky for a layer of carbyne nanocrystals, ohmic for simulations of a single carbyne chain) have been reported, which suggests that technology of carbyne layer formation will affect the contact resistance and barrier height. In either case, a good adhesion between Au and the carbyne layers is expected. As for Ag, it has been shown by calculations that the expected contact is ohmic, as even more electrons are donated from the Ag to the carbyne chain than from Au to the carbyne chain, despite the great energy level misalignment. However, the stability of the Ag-terminated carbyne chains is poor, and future work should be focused on increasing the stability of the carbyne layer first, if Ag metallization is needed. By calculations, it has been shown that Ni also donates electrons to the carbyne chain, and that should also result in an ohmic contact, alongside affecting the charge distribution in the carbyne chain. A dependence of the bond length between Ni and C of the carbyne chain length has been reported for chains with odd numbers of C atoms, where with increases of the number of carbon atoms, the bond length between Ni and C elongates. For Cu, it has been shown that by deposition of carbyne over a Cu substrate, the interaction between the carbyne chains is increased, with hypothesized Van der Waals interaction, which should improve the cohesion of the carbyne layer. As for Pt, similar to Ni, shortening of the bonds between the Pt and carbon atoms in Pt-terminated carbyne with an increase in the chain length has been reported.

Table 1 summarizes reported measurements and simulations for M–Cx–M systems, where M is metal, and C_x_ is carbyne with x number of atoms.

## 4. Conclusions

The interfaces between the carbyne and different metal films, serving as electrodes, are still unexplored. As different output signals (for example, change in resistance or capacitance) are expected from the different types of devices (transducers, sensors, etc.), the engineering of the metal contacts connecting the active layers and the signal processing circuit is crucial. It will ensure that the electrodes are not a limiting factor for the device’s performance, considering the resistance, contact energy barrier height, adhesion, and bonding ability at the packaging.

This review is on experimental and computational reports on the interactions between metals and carbyne chains, to serve as a starting guidance for the metallization of carbyne layers. The substrates on which the carbyne layers are deposited affect the stability of the carbyne, so the deposition of metal layers, which will serve as electrodes in advanced nanodevices, can lead to more stable carbyne layers. The results showed that the structures of the carbyne layer and the metal layers affect the type of contact between them. It means that the interface is dependent on the deposition technology of the layers, and on the order of carbyne growth (carbyne deposition first and then the metal film, or vice versa). It is demonstrated that the temperature can be a limiting factor for the technology of the carbyne-based devices because at higher temperatures, the carbyne undergoes structural changes. The effect of the temperature is also important at the wire bonding step of the final device in a package, so it should not be performed by heating the whole substrate, and measures must be taken to prevent overheating, such as adding multiple metal layers to dissipate the heat concentrated on the wire bonding spot by ultrasound and pressure of the wire bonding needle. It is recommended that gold, platinum, and nickel are applied in sensing structures where the existence of ohmic contact is in the fundament of the sensing mechanism, as, for example, surface acoustic wave-based sensors. Silver and copper would be preferable in transistor-based or capacitive sensing structures, where the capacitance–voltage characteristic is dependent on the threshold voltage, forming due to the Schottky contacts.

It would be beneficial for the precision of the carbyne-based sensor if the voltage drop and electrical losses at the interface with the metal contacts are minimized. Improvement of the sensor characteristics is expected, if the contacting materials are similar—for example, if organic/organic interface replaces the metal–organic interface. This should decrease the energy barrier’s height at the junction, and reduce the differences in the thermal, electrical, and mechanical properties between the sensing film and the contact electrode film.

As a perspective for future work, the effect of the contact geometry and contact edge effects on the charge transfer between the metals and carbyne at the interface, and on the charge distribution in the carbyne itself, should be explored more intensively. Additionally, the dependence of the electrical characteristics on the defects induced in the carbyne layers should be investigated, and the degree of affecting the transport properties of carbyne should be evaluated.

## Figures and Tables

**Table 1:** Bond lengths and Raman peaks for different M–C_x_–M systems.

Metal	Au	Ag	Ni		Cu	Pt
Bond length	Not reported	Not reported	x-odd 1.67 Å (x = 1) to 1.78 Å (x = 9)	x-even 1.81 Å	Not reported	2.039 Å (x = 4) 2.031 Å (x = 26)
Raman peaks	2130 cm^−1^	1985 cm^−1^ and 2117 cm^−1^.	Not reported		1960 cm^−1^ and 2100 cm^−1^	Not reported

## Data Availability

The data presented in this review are available on request from the corresponding authors of the original papers.
